# Supplementary material for: Identification of cross-reactive vaccine antigen candidates in Gram-positive ESKAPE pathogens through subtractive proteome analysis using opsonic sera
Source: PLoS One. 2025 Mar 26;20(3):e0319933. doi: 10.1371/journal.pone.0319933 (PMC11940424; doi:10.1371/journal.pone.0319933)
Supplement: S1 Data — (PDF) [file pone.0319933.s002.pdf]

Supporting information – Data sets

Identification of cross-reactive vaccine antigen candidates in Gram-positive ESKAPE pathogens through subtractive proteome analysis using opsonic sera

Océane Sadones[1], Eliza Kramarska[2], Maite Sainz-Mejías[3], Rita Berisio[2], Johannes Huebner[1*], Siobhán McClean[3], and Felipe Romero-Saavedra[1]

1. Division of Pediatric Infectious Disease, Hauner Children's Hospital, LMU, Munich, Germany.
2. Institute of Biostructures and Bioimaging, Italian Research Council (CNR), Naples, Italy.
3. School of Biomolecular and Biomedical Sciences and UCD Conway Institute of Biomedical and Biomolecular Research, University College Dublin, Belfield, Dublin 4 D04 V1W8, Ireland.

*Correspondence: johannes.huebner@med.uni-muenchen.de

Figure 2C

	Human sera			Depleted Human Sera		
**Dilution factor**	**10^2**	**10^4**	**10^6**	**10^2**	**10^4**	**10^6**
**Killing (%)**	55,4	22,0	17,3	38,8	14,1	6,3
**Killing (%)**	63,3	27,0	21,8	38,8	11,1	8,9
**Killing (%)**	76,4	24,2	17,1	45,4	10,1	11,4
**Killing (%)**	54,1	21,5	12,6	45,4	5,2	3,8
**Mean**	62,3	23,7	17,2	42,1	10,1	7,6

Figure 2D

	Human sera			Depleted Human Sera		
**Dilution factor**	**10^2**	**10^4**	**10^6**	**10^2**	**10^4**	**10^6**
**Killing (%)**	50,6	29,2	0,1	30,4	17,8	2,0
**Killing (%)**	55,6	31,0	10,9	36,8	15,6	9,8
**Killing (%)**	39,1	23,9	3,5	35,2	24,4	1,1
**Killing (%)**	42,4	27,4	1,7	27,3	20,0	0,5
**Mean**	46,9	27,9	4,0	32,4	19,4	3,4

Figure 5A

	Pre-$AdcA_{au}$			Anti-$AdcA_{au}$		
**Concentration µg/ml**	**0,5**	**0,25**	**0,125**	**0,5**	**0,25**	**0,125**
**Abs_{405nm}**	0,093	0,086	0,082	4,000	2,887	1,732
**Abs_{405nm}**	0,094	0,088	0,081	3,999	3,340	1,985
**Abs_{405nm}**	0,091	0,087	0,083	4,000	3,111	1,857
**Abs_{405nm}**	0,095	0,087	0,080	4,000	3,117	1,861
**Mean**	0,094	0,087	0,082	4,000	3,114	1,859

Figure 5B

	Pre-AdcA$_{au}$			Anti-AdcA$_{au}$		
**Concentration µg/ml**	**60**	**30**	**15**	**60**	**30**	**15**
**Abs$_{405nm}$**	0,104	0,098	0,099	1,973	1,361	0,866
**Abs$_{405nm}$**	0,103	0,117	0,089	2,402	1,653	1,192
**Abs$_{405nm}$**	0,100	0,094	0,088	2,180	1,487	0,886
**Abs$_{405nm}$**	0,098	0,093	0,090	2,196	1,527	1,172
**Mean**	0,101	0,101	0,092	2,188	1,507	1,029

Figure 6A

	Pre-AdcA$_{au}$	Anti-AdcA$_{au}$			
**Dilution**	**40**	**40**	**80**	**160**	**320**
**Killing (%)**	2,2	37,2	28,6	15,2	22,6
**Killing (%)**	4,2	28,2	38,0	26,2	29,0
**Killing (%)**	0,2	40,6	25,4	26,5	9,7
**Killing (%)**	7,6	43,3	22,5	36,4	19,4
**Mean**	3,6	37,3	28,6	26,1	20,2

Figure 6B

	Anti-AdcA$_{au}$				
**Inhibitor (µg/ml)**	**0**	**200**	**100**	**40**	**8**
**Killing (%)**	50,3	7,8	27,5	34,1	38,2
**Killing (%)**	53,0	8,6	22,3	43,6	47,6
**Killing (%)**	48,9	4,6	33,5	34,1	53,0
**Killing (%)**	32,8	14,4	31,5	36,8	43,6
**Mean**	46,2	8,8	28,7	37,2	45,6

Figure 7

	S. aureus Reynolds 1:20		*E. faecium 11236/1* 1:20		*E. faecalis 12030* 1:50		*E. faecalis Type 2* 1:10	
**Serum**	**Pre-AdcA$_{au}$**	**Anti-AdcA$_{au}$**	**Pre-AdcA$_{au}$**	**Anti-AdcA$_{au}$**	**Pre-AdcA$_{au}$**	**Anti-AdcA$_{au}$**	**Pre-AdcA$_{au}$**	**Anti-AdcA$_{au}$**
**Killing (%)**	21,1	49,5	18,5	54,9	37,8	79,8	19,4	48,7
**Killing (%)**	26,3	48,6	21,5	66,9	35,0	78,3	3,3	48,7
**Killing (%)**	33,1	49,5	32,1	51,9	43,3	73,6	24,4	48,7
**Killing (%)**	22,9	49,2	16,5	51,9	35,0	82,9	7,3	55,9
**Mean**	25,9	49,2	22,2	56,4	37,8	78,7	13,6	50,5
